# Muscle-specific deletion of *Arid5b* causes metabolic changes in skeletal muscle that affect adipose tissue and liver

**DOI:** 10.3389/fendo.2022.1083311

**Published:** 2023-01-18

**Authors:** Jennifer Murray, Ali Ehsani, Liza Najjar, Guoxiang Zhang, Keiichi Itakura

**Affiliations:** Center for RNA Biology and Therapeutics, Beckman Research Institute, City of Hope, Duarte, CA, United States

**Keywords:** Arid5b, skeletal muscle, glucose, fatty acids, metabolism

## Abstract

Emerging evidence suggests that AT-Rich Interaction Domain 5b (Arid5b) may play a role in energy metabolism in various tissues. To study the metabolic function of Arid5b in skeletal muscle, we generated skeletal muscle-specific *Arid5b* knockout (Arid5b MKO) mice. We found that Arid5b MKO skeletal muscles preferentially utilized fatty acids for energy generation with a corresponding increase in FABP4 expression. Interestingly, in Arid5b MKO mice, the adipose tissue weight decreased significantly. One possible mechanism for the decrease in adipose tissue weight could be the increase in phospho-HSL and HSL expression in white adipose tissue. While glucose uptake increased in an insulin-independent manner in Arid5b MKO skeletal muscle, glucose oxidation was reduced in conjunction with downregulation of the mitochondrial pyruvate carrier (MPC). We found that glucose was diverted into the pentose phosphate pathway as well as converted into lactate through glycolysis for export to the bloodstream, fueling the Cori cycle. Our data show that muscle-specific deletion of Arid5b leads to changes in fuel utilization in skeletal muscle that influences metabolism in other tissues. These results suggest that Arid5b regulates systemic metabolism by modulating fuel selection.

## Introduction

Skeletal muscle is an important regulator of energy balance and is responsible for a major portion of whole-body glucose and lipid utilization. Metabolic flexibility in response to fasting or exercise is crucial to maintaining energy homeostasis. Perturbations in glucose and lipid metabolism have been linked to diseases such as diabetes (1) and cancer (2). Skeletal muscle is responsible for a majority of insulin-stimulated glucose uptake, and therefore is a critical component of whole-body insulin resistance (3). In skeletal muscle, insulin resistance has been associated with the dysregulation of lipid metabolism, including changes in fatty acid uptake, fatty acid oxidation, and triacylglyceride (TG) synthesis and breakdown (4). Additionally, defects in insulin signaling have been linked to the buildup of lipid intermediates, such as diacylglycerols, ceramides, and fatty acyl-CoAs, along with an increase in circulating TGs and fatty acids (5). Therefore, changes in fatty acid metabolism in skeletal muscle can promote the development of insulin resistance and diabetes.

The AT-Rich Interaction Domain (ARID) family is comprised of 7 subfamilies, ARID1-5 and jumonji ARID 1 (JARID1) and JARID2 (6). All family members contain the ARID helix-turn-helix DNA binding domain. Arid5b is widely expressed in many tissues (7), and ARID family members have been shown to regulate differentiation and gene expression in cells of the mesenchymal lineage (8). Previous studies have also suggested that Arid5b may have a role in metabolism in diverse tissues. Whole-body deletion of Arid5b resulted in reduced adipose tissue size and resistance to weight gain on a high-fat diet (9). Knockdown of Arid5b was shown to inhibit adipogenesis and increase fatty acid recycling in 3T3-L1 cells (10, 11). In hepatocytes phosphorylation of PHF2 by PKA led to the formation of a PHF2-Arid5b complex on the promoters of *Pepck* and *G6Pase*, leading to removal of histone repressor marks on these promoters (7). In human CMV-associated natural killer cells, upregulation of Arid5b enhanced mitochondrial oxidative metabolism, mitochondrial membrane potential and expression of genes encoding components of the electron transport chain (12).

We previously demonstrated that Arid5b promotes skeletal muscle differentiation by regulating prostaglandin I production (13). We also showed that glucose metabolism was enhanced in the skeletal muscle of whole-body Arid5b^-/-^ mice (14). However, to more fully elucidate the function of Arid5b in a tissue-specific manner, we generated skeletal-muscle specific Arid5b (MKO) knockout mice. Fatty acid oxidation was increased in Arid5b MKO skeletal muscle accompanied by increased FABP4 expression. Unlike the Randle cycle, in which increased fatty acid oxidation leads to a decrease in glucose utilization, we found that glucose uptake was enhanced but glucose oxidation was decreased in Arid5b MKO skeletal muscle. Imported glucose was metabolized by the pentose phosphate pathway and through glycolysis to lactate. Intriguingly, these metabolic changes in the skeletal muscle of Arid5b MKO mice influenced the weight reduction in adipose tissue and Cori cycling in the liver.

## Materials and methods

### Animal studies

All animal experiments were approved by the City of Hope Institutional Animal Care and Use Committee under protocol 13044. MyoD^iCre^ mice (FVB.Cg-*MyoD1^tm2.1(icre)Glh^*, Stock #014140) (15) were obtained from The Jackson Laboratory (Bar Harbor, ME) and contain an optimized Cre recombinase knocked-in to the first exon of the *MyoD* gene. The MyoD^iCre^ mice, which were generated using the FVB strain, were backcrossed to C57BL/6 for 6 generations using the Speed Congenics services from The Jackson Laboratories to generate mice that were >99% C57BL/6. These MyoD^iCre^ mice were then crossed with Arid5b^flox^ mice (16), which contain LoxP sites flanking exon 6 of *Arid5b*, to generate the *Arid5b* muscle-specific knockout mice (Arid5b MKO). Mice had free access to standard chow and water and were maintained under a 12h light-dark cycle. 12-14 week-old male mice were used for all experiments. Respiratory quotient, energy expenditure and movements were analyzed at the City of Hope Comprehensive Metabolic Phenotyping Core using the Promethion Metabolic Caging System (Sable Systems, North Las Vegas, NV).

### Glucose tolerance tests

Mice were fasted for 7-8 hours. Blood glucose and lactate levels were measured from the tail using the Clarity Diagnostics (Boca Raton, FL) BG1000 blood glucose meter and the Lactate Plus lactate meter (Nova Biomedicals, Waltham, MA) at 0, 10, 20, 30, 60 and 120 minutes after intraperitoneal glucose injection (1g/kg body weight).

### Quantitative real-time PCR

Tissues were homogenized using a Polytron PT 2500 E (Kinematica, Bohemia, NY) in QIAzol (Qiagen, Germantown, MD, USA), and RNA was isolated using the miRNeasy kit (Qiagen). RNA was DNase treated using the RNase-free DNase set (Qiagen). Reverse transcription was carried out using the iScript cDNA synthesis kit (Bio-Rad, Hercules, CA, USA). Real-time PCR reactions contained 1X SYBR Green reagent (Bio-Rad) and 0.1µM gene specific primers. Experimental transcript levels were analyzed on the CFX96 real-time PCR system (Bio-Rad). Results were normalized to Rpl19 expression analyzed in separate reactions. Mitochondrial DNA analysis was performed as previously described (17).

### Immunoblot analysis

Skeletal muscles were homogenized using a Polytron PT 2500 E (Kinematica) in TNS buffer (20mM Tris, 50mM NaCl, and 250mM sucrose) containing 1mM DTT, 0.5mM PMSF, and 1x Halt™ Protease and Phosphatase Inhibitor (Thermo Fisher Scientific). Samples were solubilized by addition of Triton X-100 (1%) for 1 h at 4°C on a rotator. Lysates were spun at 7,000 x g for 5 min at 4°C, and the supernatant was collected. Adipose tissues were homogenized in buffer containing 150mM NaCl, 50mM Tris-HCl, pH 7.6, and 5mM EDTA with 1mM PMSF and 1x Halt™ Protease and Phosphatase Inhibitor. Homogenates were spun at 5,000 x g for 5min at 4°C, and the supernatant was collected avoiding the upper fat layer. This centrifugation step was repeated, and then Triton X-100 was added to a final concentration of 1%. Samples were rotated for 1 h at 4°C, spun at 14,000 x g at 4°C for 15min, and supernatants were collected. Protein concentration was determined with the Pierce BCA protein assay (Thermo Fisher Scientific). Proteins were resolved by SDS-PAGE and transferred to nitrocellulose membranes (Bio-Rad). Membranes were blocked in 5% non-fat milk, and primary antibody incubations were performed overnight at 4°C. The following primary antibodies were used: P-Akt (Ser473) (Cell Signaling, Danvers, MA, USA), Akt (Cell Signaling), AS160 (Abcam, Waltham, MA), FABP4 (Cell Signaling), G6PD (Abcam), GLUT4 (Cell Signaling), HSP70 (Cell Signaling), HSP90 (Cell Signaling), P-HSL (Ser660) (Cell Signaling), HSL (Cell Signaling), MPC1 (Cell Signaling), MPC2 (Cell Signaling), PGC-1α (Thermo Fisher Scientific), TBC1D1 (Cell Signaling), Total OXPHOS Rodent WB antibody cocktail (Abcam), β-tubulin (Cell Signaling), VDAC1 (Abcam), and vinculin (Cell Signaling). After incubation with horseradish peroxidase-labeled secondary antibodies, proteins were visualized with ProSignal DURA ECL reagent (Genessee Scientific, El Cajon, CA). Images were captured on the Bio-Rad ChemiDoc MP Imaging System and quantitated with Image Lab software (Bio-Rad).

### Coimmunofluorescence staining

Coimmunofluorescence staining for GLUT4 and dystrophin on GC and Sol muscle sections and calculation of the Pearson’s correlation coefficient were performed as described previously (14).

### Plasma triglycerides and free fatty acids

Blood was collected by cardiac puncture and put into tubes containing EDTA. Samples were spun at 1,000 x g for 10 min at 4°C, and plasma was collected. Triglycerides in the plasma were measured using the High Sensitivity Triglyceride Assay Kit (Sigma, St. Louis, MO) according to the manufacturer’s instructions. Free fatty acids were measured with the Free Fatty Acid Quantification Assay Kit (Abcam) according to the manufacturer’s instructions.

### Glycogen assay

Glycogen content in the liver was measured using the Glycogen Assay Kit II (Colorimetric) (Abcam) according to the manufacturer’s instructions.

### Glucose and fatty acid oxidation assays

For glucose oxidation assays, isolated skeletal muscles were incubated in Krebs Henseleit Bicarbonate (KHB) buffer (116 mM NaCl, 4.6 mM KCl, 1.16 mM KH_2_PO_4_, 25.3 mM NaHCO_3_, 2.5 mM CaCl_2_, 1.16 mM MgSO_4_, 2 mM glucose, 38 mM mannitol and 2% fatty-acid free BSA) containing 1 µCi/ml [U-^14^C]-D-glucose (American Radiolabeled Chemicals, St. Louis, MO) and 50 µM palmitate at 37°C with 5% CO_2_ for 1 hr in a sealed vial containing a separate tube of benzethonium hydroxide to collect ^14^CO_2_. At the end of the incubation, 100 µl of 60% perchloric acid was injected into each vial. The vials were resealed and incubated overnight at 4°C. The tube containing the benzethonium hydroxide was then transferred to a new vial, scintillation fluid was added to each vial, and radioactivity was counted on a Beckman Coulter LS6500 liquid scintillation counter. Data were normalized to tissue weights. For fatty acid oxidation assays, isolated skeletal muscles were incubated with 125 µM [^3^H-9, 10]-palmitic acid (Perkin Elmer) and 1mM carnitine for 2 hr at 37°C with 5% CO_2_. ^3^H_2_O released from FAO was separated over Dowex 1 X 2-400 anion exchange resin (Sigma) and quantitated by scintillation counting. Data were normalized to tissue weights.

### Metabolomics

For each muscle type, samples were pooled from 4 individual Arid5b WT or 4 Arid5b MKO mice. For metabolites extraction, 15 mg of biopulverized tissue was resuspended in ice-cold methanol: acetonitrile: water (2:1:1, v/v/v) containing four internal standards (d_8_-Valine, ^13^C_6_-Phenyl alanine, ^13^C_6_-adipic acid, d_4_-succinic acid (18, 19) and subjected to 5 bead-beating cycles with 1 min on ice between each cycle. The homogenate was centrifuged at 25,000g for 10 min at 4°C, and the supernatant was vacuum-dried and preserved for metabolite analysis. Targeted analysis on ATP and pyruvate was performed using a Vanquish UPLC and TSQ-Altis triple quadrupole mass spectrometer (Thermo Fisher), as described previously (20). Parallel Reaction Monitoring (PRM) of lactate was performed on metabolite extracts from biopulverized mouse tissues spiked with 15µL of [U13C] yeast metabolite extract (Cambridge Isotope laboratories, Andover, MA). The HILIC chromatography with solvent A (10mM ammonium acetate, 0.1% ammonium hydroxide in 95% Water, 5% ACN) and solvent B (acetonitrile in 0.05% ammonium hydroxide) on a BEH amide column with a flow rate of 0.4 mL/min and a 15 min gradient (99% B for 1 min, 99% to 85% for 2 min, 85% to 75% B for 3 min, 75% to 30% B for 3 min, 30% B for 1 min) was used for metabolite separation. MS1 and PRM data were acquired on an Orbitrap Eclipse tribrid mass spectrometer (Thermo) with the mass range of 300 to 1500 *m/z* in positive mode and 50 to 200 *m/z* in negative mode in orbitrap. MS1 and MS2 targeted data extraction was performed using Skyline 20.1.0.155. All analytes of interest were within limits of acceptance (area of more than 75% of analytes were within 25% CV) in the quality control samples. Across all injections, analytes were within ±5ppm mass error and ±2min retention time deviation. [U13C] lactate was below the limit of quantitation. Both MS1 precursor ion and MS2 product ions were used for relative quantitation.

### Statistical analysis

Significant differences between groups were determined by unpaired Student’s *t* test, with a value of P ≤ 0.05 considered significant.

## Results

### Generation and characterization of Arid5b muscle-specific knockout mice

**Figure 1:**
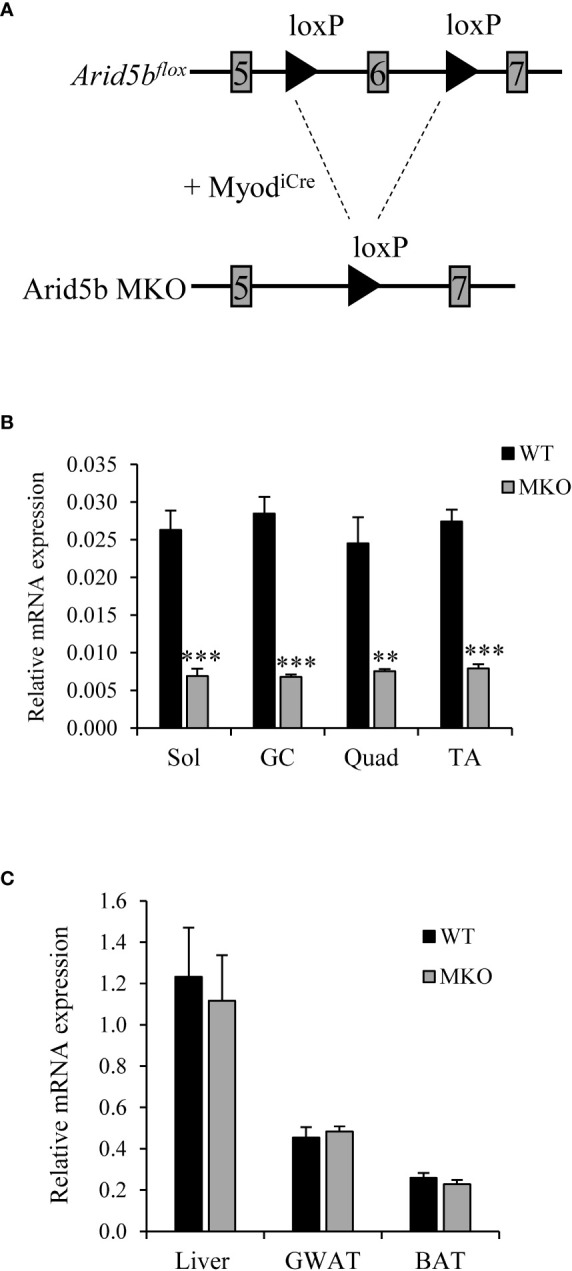
Arid5b is deleted specifically in skeletal muscles. **(A)** Diagram of the knockout strategy used to generate skeletal muscle-specific Arib5b knockout mice. **(B)** Expression levels of Arid5b in soleus (Sol), gasctrocnemius (GC), quadriceps (Quad), and tibialis anterior (TA) skeletal muscles were determined by quantitative real-time PCR and normalized to Rpl19 mRNA levels. Samples were analyzed in triplicate, and data are expressed as the means ± SE. **, p < 0.01; ***, p < 0.001. (n = 4-8). **(C)** Arid5b expression levels were analyzed by real-time PCR in liver, GWAT and BAT and normalized to Rpl19 mRNA levels. Samples were analyzed in triplicate, and results are expressed as the means ± SE. (n = 6-9).

To investigate the function of Arid5b in skeletal muscle, we generated skeletal-muscle specific Arid5b knockout (Arid5b MKO) mice using MyoD-iCre knockin mice (**Figure 1A**). *Arid5b^flox^* mice, which contain LoxP sites flanking exon 6 of the *Arid5b* gene, were mated with heterozygous Myod^iCre^ mice in which the *iCre* recombinase is knocked in to exon 1 of the *MyoD* gene (21). *MyoD* expression is restricted to skeletal muscle lineage cells and therefore selectively knocks out *Arid5b* in skeletal muscles. *Arid5b* transcript expression was decreased in the skeletal muscles of Arid5b MKO mice (**Figure 1B**) but not in non-muscle tissues such as white adipose, brown adipose, and liver (**Figure 1C**).

**Figure 2:**
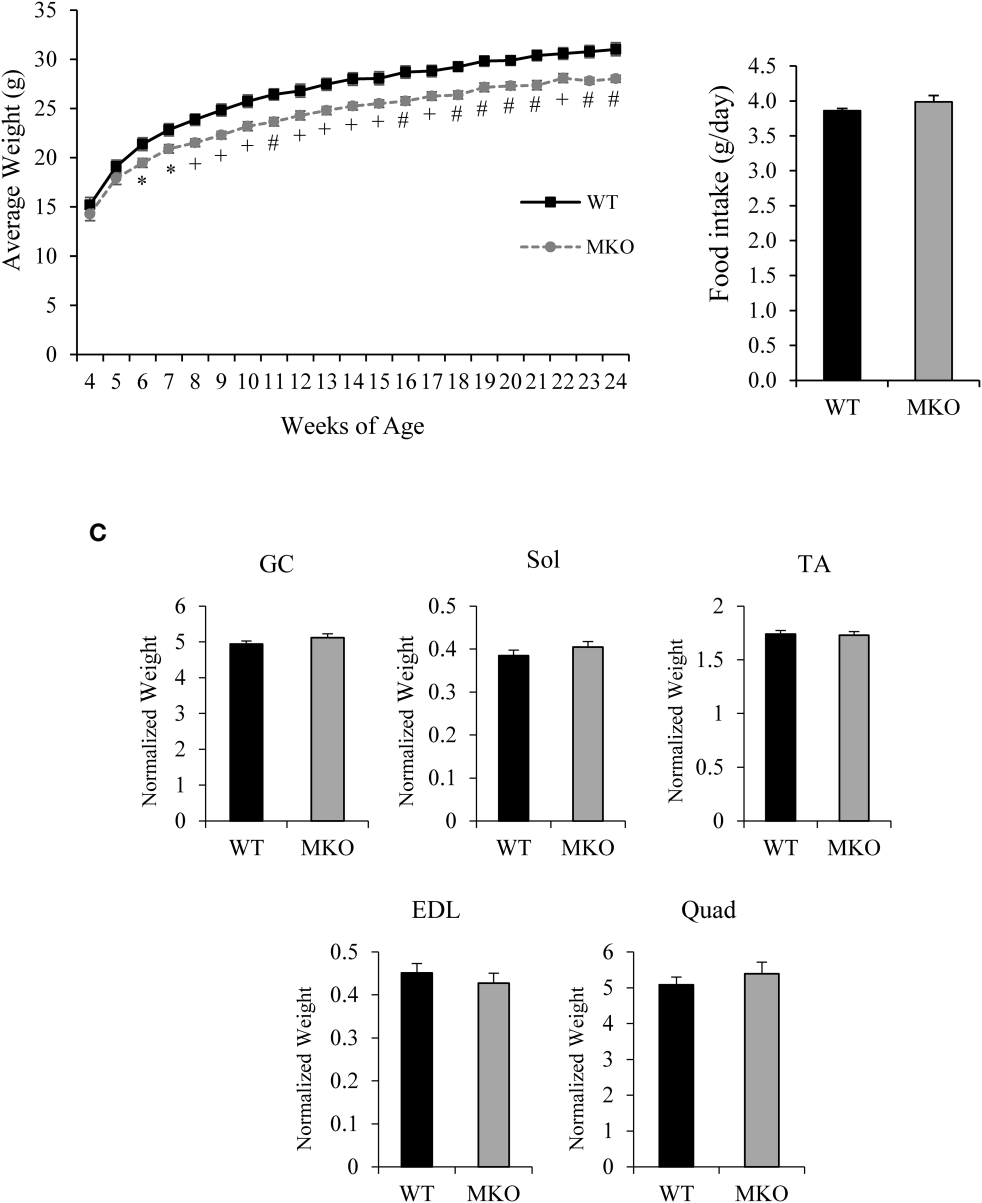
The body weight of Arid5b MKO mice was reduced relative to WT mice while food intake and skeletal muscle weights were similar for Arid5b WT and MKO mice. **(A)** Growth curve of Arid5b WT and MKO mice (n=10). *, p < 0.05; +, p < 0.01; #, p < 0.001. **(B)** Food intake of Arid5b WT and MKO mice expressed as the means ± SE (WT, n=7; MKO, n=8). **(C)** Skeletal muscle weights were normalized to body weight, and results are expressed as the means ± SE. (WT, n=14; MKO, n=11).

The body weight of Arid5b MKO mice was significantly reduced by 2.6 ± 0.3 g relative to Arid5b WT mice beginning at 6 weeks of age (**Figure 2A**). The significant weight loss was not due to changes in food consumption between Arid5b WT and Arid5b MKO mice (**Figure 2B**). To determine if the reduction in body weight of the Arid5b MKO mice was due to a decrease in skeletal muscle mass, we analyzed the weights of skeletal muscles normalized to body weight. We found no significant difference in the weights of gastrocnemius (GC), soleus (Sol), tibialis anterior (TA), extensor digitorum longus (EDL), or quadriceps (Quad) between Arid5b WT and Arid5b MKO mice (**Figure 2C**). We then performed comprehensive metabolic phenotyping analysis to assess locomotor activity, fuel utilization and metabolic rate. We found that the distance traveled, respiratory quotient, and energy expenditure were similar between Arid5b WT and MKO mice (**Supplementary Figure 1**). This characterization data suggests that the weight loss in Arid5b MKO mice was not due to changes in skeletal muscle mass after Arid5b deletion.

### Arid5b MKO mice show increased fatty acid oxidation in skeletal muscles and reduction of adipose tissue mass

**Figure 3:**
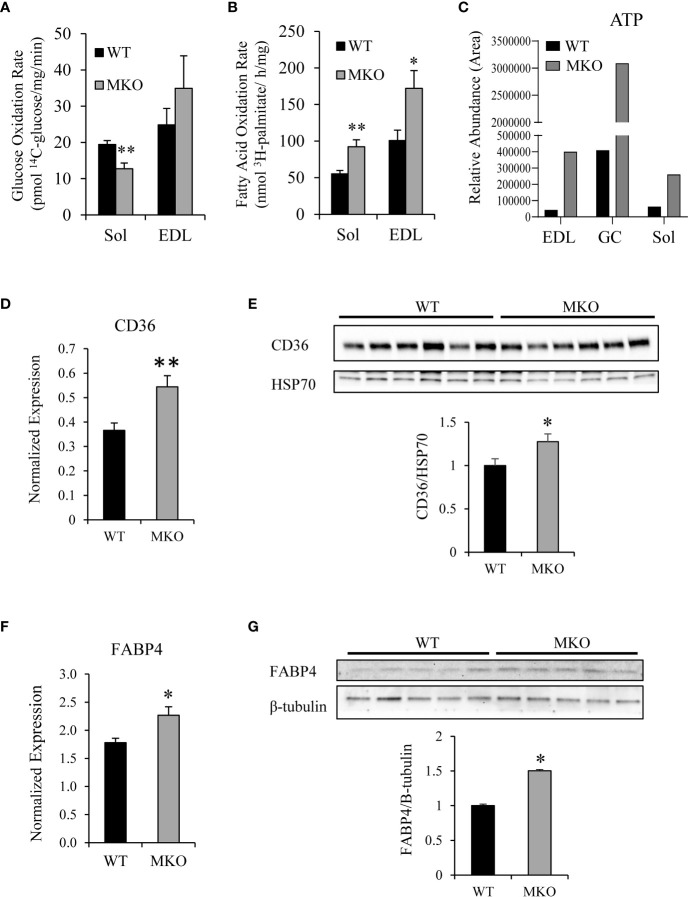
Arid5b MKO skeletal muscles preferentially utilize fatty acids. **(A)** Glucose oxidation rates were determined in isolated skeletal muscles by measuring ^14^CO_2_ release after incubation with ^14^C-glucose. (Sol, n=6; EDL, n=5). **, p < 0.01. **(B)** Palmitate oxidation rates were determined in isolated skeletal muscles by measuring ^3^H_2_O release after incubation with ^3^H-palmitic acid. (Sol, n=6; EDL, n=5) *, p < 0.05; **, p < 0.01. **(C)** Mass spectrometry analysis of ATP content was performed in Arid5b WT and MKO skeletal muscles. **(D)** CD36 expression was analyzed by real-time PCR and normalized to Rpl19 expression. Samples were analyzed in triplicate, and results are expressed as the means ± SE (n=7-8). **, p < 0.01. **(E)** Western blot analysis was performed for CD36 expression in GC muscles and normalized to HSP70 expression. Quantitation of CD36 expression was performed, and data are presented as the means ± SE. *, p < 0.05. **(F)** FABP4 expression was analyzed by real-time PCR and normalized to Rpl19 expression. Samples were analyzed in triplicate, and results are expressed as the means ± SE (n=8). *, p < 0.05. **(G)** Western blot analysis was performed for FABP4 expression in GC muscles and normalized to β-tubulin expression. Quantitation of FABP4 expression was performed, and data are presented as the means ± SE. *, p < 0.05.

While the metabolic phenotyping analysis measures metabolic parameters at the whole-body level, these changes may be subtle in a tissue-specific knockout model system. Therefore, to determine the substrate utilization profile of Arid5b MKO skeletal muscles, we assessed the rates of glucose oxidation and fatty acid oxidation in isolated skeletal muscles. We found that the rates of glucose oxidation were decreased by 34% in Arid5b MKO Sol compared to Arid5b WT muscles (**Figure 3A**). Interestingly, the fatty acid oxidation rates were increased in Arid5b MKO Sol and EDL by 1.7-fold relative to Arid5b WT muscles (**Figure 3B**). Consistent with the increased rates of fatty acid oxidation, targeted mass spectrometry analysis revealed that ATP levels were increased in Arid5b MKO muscles (**Figure 3C**), suggesting that the Arid5b MKO mice produced excess ATP that accumulated in the skeletal muscles. Additionally, energy expenditure did not change between Arid5b WT and MKO mice (**Supplementary Figure 1**). An increase in fatty acid oxidation suggests that the expression of fatty acid transporters may also be elevated. Therefore, we analyzed the expression of carnitine palmitoyltransferase 1b (Cpt1b), which imports fatty acids into the mitochondria (22), cluster of differentiation 36 (CD36), which is one of the major importers of fatty acids into the cell (23), and fatty acid binding protein 4 (FABP4), which is involved in fatty acid uptake and transport in skeletal muscle (24, 25). While the expression of Cpt1b did not change between Arid5b WT and MKO GC (**Supplementary Figure 2**), CD36 expression was significantly upregulated in Arid5b MKO GC at both the RNA (**Figure 3D**) and protein levels (**Figure 3E**). Gene expression analysis also showed that FABP4 was upregulated in Arid5b MKO GC relative to Arid5b WT transcriptionally (**Figure 3F**) and translationally (**Figure 3G**). Taken together, these results indicate that there is a substrate switch in Arid5b MKO muscles favoring the utilization of fatty acids rather than glucose for energy generation.

**Figure 4:**
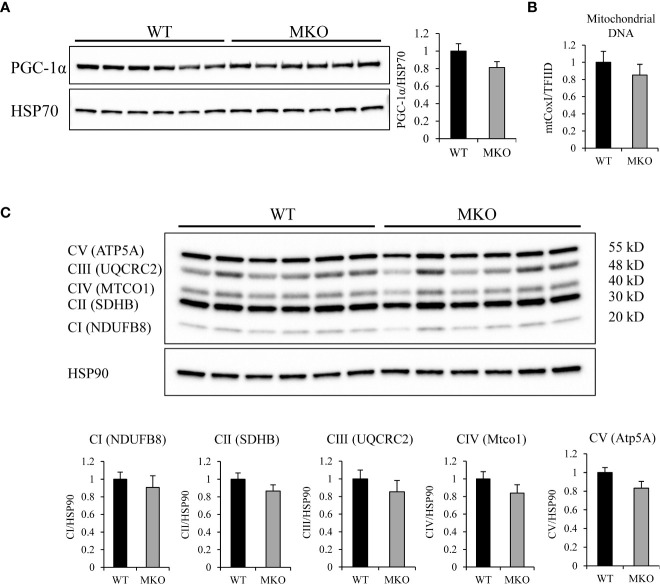
Expression of PGC-1α and ETC complexes and mitochondrial DNA analysis in Arid5b WT and MKO skeletal muscle. **(A)** Western blot analysis was performed for PGC-1α expression in GC muscles. HSP70 was included as a loading control. Quantitation of PGC-1α expression was performed, and data are presented as the means ± SE. **(B)** To assess mitochondrial content, DNA was isolated from GC muscles. Real-time PCR was performed for the mitochondrial-encoded gene *mtCox1*and the nuclear encoded gene *TFIID*, and mitochondrial content was assessed from the ratio of CoxI to TFIID (n=4). Samples were analyzed in triplicare, and results are expressed as the means ± SE. **(C)** (top) Western blot analysis was performed for expression of five ETC subunits in GC muscles. HSP90 was included as a loading control. CI: NADH:ubiquinone oxidoreductase subunit B8 (NDUFB8), CII: succinate dehydrogenase complex iron sulfur subunit B (SDHB), CIII: ubiquinol-cytochrome C reductase core protein 2 (UQCRC2), CIV: mitochondrially encoded cytochrome C oxidase I (MTCO1), CV: ATP synthase, H+ transporting, mitochondrial F1 complex, alpha subunit 1 (ATP5A1). (bottom) Quantitation of each subunit was performed, and data are presented as the means ± SE.

To determine if the substrate switch towards fatty acids was due to an increase in mitochondrial biogenesis, we analyzed the expression of PGC-1α, components of the mitochondrial electron transport chain (ETC), and mitochondrial DNA content to quantitatively measure mitochondrial number. PGC-1α is an important metabolic regulator that induces mitochondrial biogenesis and oxidative metabolism (26, 27). Western blot analysis showed that the protein expression of PGC-1α (**Figure 4A**) was similar between the two genotypes. Mitochondrial DNA content was not significantly different between Arid5b WT and MKO GC (**Figure 4B**), indicating that mitochondrial number was comparable between the two genotypes. Western blot analysis for mitochondrial transcription factor A (TFAM), which is involved in mitochondrial replication (28), revealed that expression levels of this protein did not change between Arid5b WT and MKO GC (**Supplementary Figure 3**). Additionally, expression of ETC subunits were similar in Arid5b MKO GC relative to the WT GC (**Figure 4C**). All together, these results indicate that the increased fatty acid metabolism in Arid5b MKO muscles may not be due to changes in mitochondrial biogenesis.

**Figure 5:**
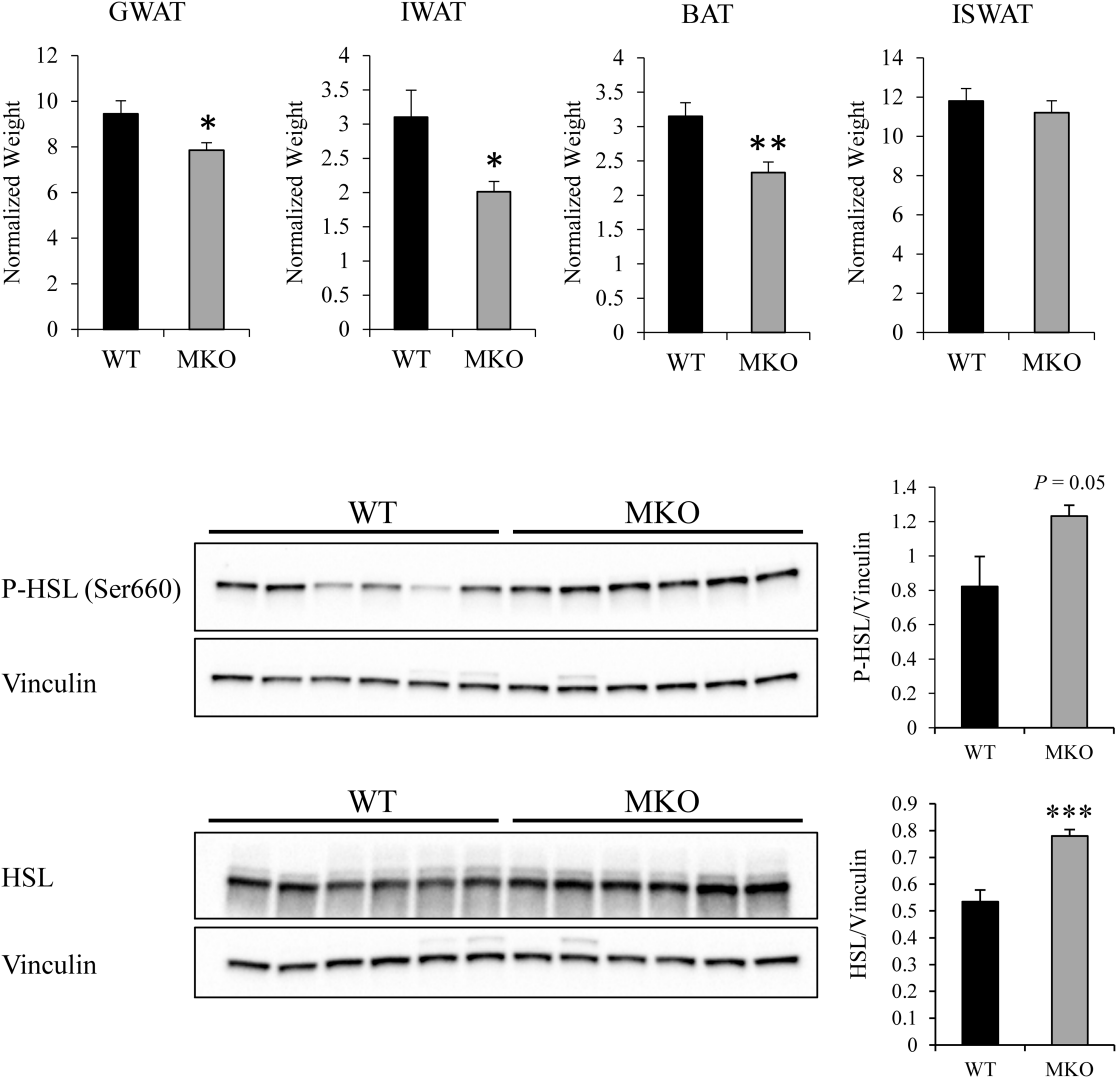
Adipose tissue size is reduced and lipolysis is increased in Arid5b MKO mice. **(A)** Adipose tissue weights were normalized to body weight, and results are expressed as themeans ± SE. (WT, n=13-14; MKO, n=8-11). *, p < 0.05; **, p < 0.01. **(B)** (right) Western blot analysis was performed for phospho-HSL (Ser660) and total HSL expression in Arid5b WT and MKO GWAT. Vinculin was included as a loading control. (left) Quantitation was performed. ***, p < 0.001.

**Figure 6:**
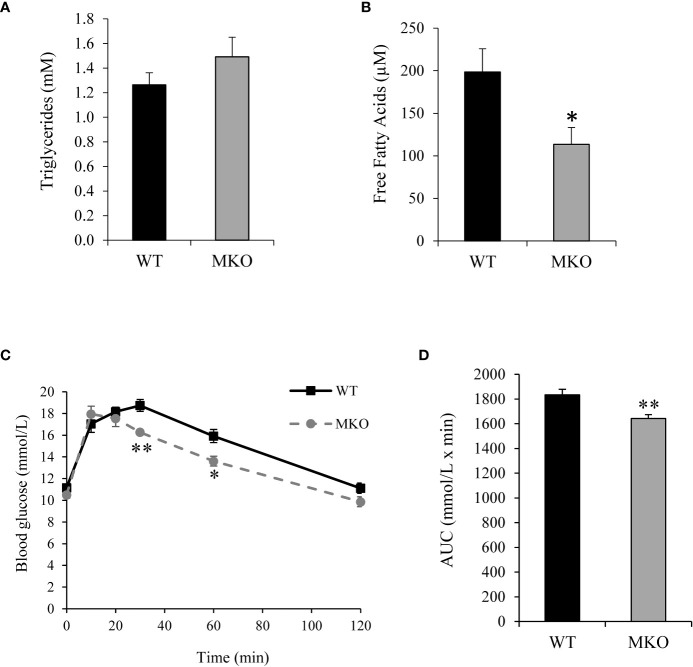
Plasma free fatty acid concentration was reduced and blood glucose clearance was increased in Arid5b MKO mice. **(A)** Triglyceride concentrations (n=8) and **(B)** free fatty acid concentrations (n=6) were analyzed in plasma from Arid5b WT and MKO mice. Data are presented as the means ± SE. *, p < 0.05. **(C)** After a 7-8 hour fast, blood glucose was measured at baseline (Time 0) and at the indicated time points after an i.p. injection of glucose. **(D)** The area under the curve was calculated. (n=6). *, p < 0.05; **, p < 0.01.

Alterations in skeletal muscle fuel consumption may influence metabolism in other organs, such as adipose tissue and liver. The majority of long-chain fatty acids are derived from lipolysis in adipose tissue, and these fatty acids are then exported to the blood stream for uptake by skeletal muscle (29). Therefore, we investigated whether there were alterations in the storage or release of fatty acids in Arid5b MKO adipose tissue. Interestingly, we found significant reductions in the normalized weights of adipose tissues, such as GWAT, IWAT, and BAT, in the Arid5b MKO mice (**Figure 5A**). Hematoxylin and eosin (H&E) staining of GWAT tissue sections revealed that adipocyte size was trending lower but did not reach significance in Arid5b MKO relative to WT (**Supplementary Figure 4**). To determine if the decrease in the adipose tissue weight was due to increased lipolysis, we performed western blot analysis for phospho-HSL (Ser660) and HSL. We found that phospho-HSL and total HSL were increased in the GWAT of Arid5b MKO mice (**Figure 5B**), indicating that lipolysis is enhanced. We then assessed the circulating free fatty acid and triglyceride content in plasma from the Arid5b WT and MKO mice. Interestingly, we found that while triglyceride levels were similar (**Figure 6A**), the free fatty acid concentration was significantly reduced in Arid5b MKO plasma relative to WT plasma (**Figure 6B**). These results indicate that the Arid5b MKO skeletal muscles take up more fatty acids from the bloodstream, consistent with the increased fatty acid oxidation rate and upregulation of FABP4. The knockout of Arid5b in the skeletal muscle revealed that there is crosstalk between the skeletal muscle and adipose tissue in Arid5b MKO mice.

### Arid5b MKO mice show increased whole-body glucose clearance, lactate export, and Cori cycling

**Figure 7:**
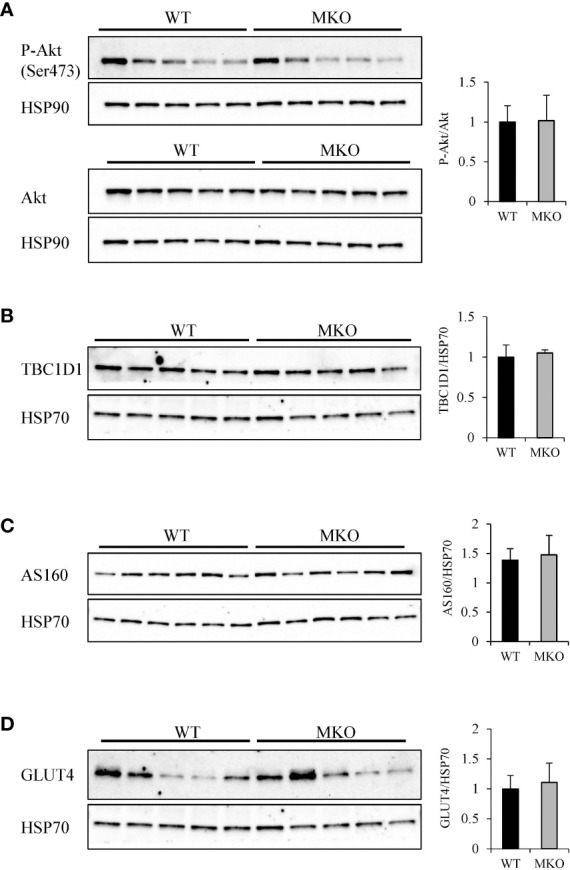
Analysis of genes regulating glucose uptake. **(A)** Western blot analysis of P-Akt (Ser473) (top) and Akt (bottom) expression in Arid5b WT and MKO GC. HSP90 was included as a loading control. Quantitation of normalized P-Akt to normalized total Akt is shown on the right. The expression of **(B)** TBC1D1, **(C)** AS160, and **(D)** GLUT4 were analyzed by Western blot in Arid5b WT and MKO GC and normalized to HSP70. Quantitation was performed, and results are expressed as the means ± SE.

**Figure 8:**
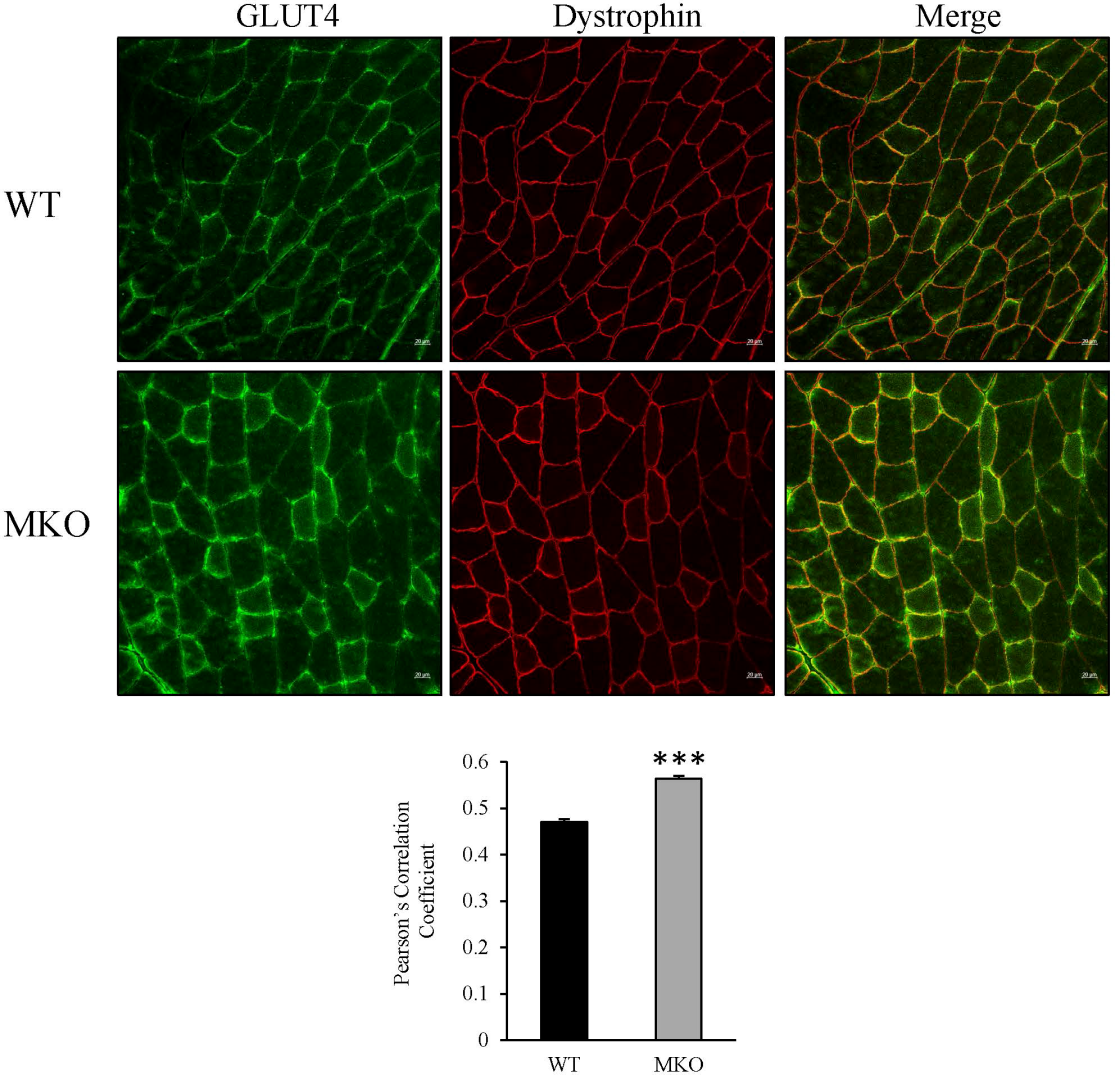
GLUT4 localization to the plasma membrane was increased in Arid5b MKO GC. Coimunofluorescence analysis for GLUT4 (green) and dystrophin (red) was carried out in GC tissue sections. Representative confocal images are shown. Scale bars = 20µm. Merged images show increased colocalization of GLUT4 and dystrophin at the plasma membrane in Arid5b MKO GC relative to WT GC. Pearson’s correlation coefficient was calculated, and data are presented as the means ± SE. ***, p < 0.001. (n=4).

To determine how skeletal muscle-specific ablation of Arid5b affects whole body glucose metabolism, we performed glucose tolerance tests (GTT). Interestingly, blood glucose clearance was increased at the 30 min and 60 min time points in Arid5b MKO mice compared to Arid5b WT mice (**Figure 6C**). Accordingly, the area under the curve was significantly reduced in Arid5b MKO mice (**Figure 6D**). The increased glucose uptake could be due to alterations in glucose transport pathways. Since Akt is an important regulator of glucose uptake, we analyzed its phosphorylation and expression levels. Western blot analysis showed that Akt Ser473 phosphorylation levels and total Akt expression levels were similar in Arid5b MKO and Arid5b WT GC (**Figure 7A**). These results suggest that the glucose uptake is insulin-independent in Arid5b MKO and not occurring through Akt or mTOR signaling. We previously reported that whole-body deletion of Arid5b expression enhanced glucose uptake and metabolism and was associated with the downregulation of TBC1D1 expression (14). However, the protein expression levels of TBC1D1 (**Figure 7B**) and its homolog, AS160, were similar in Arid5b MKO and Arid5b WT GC (**Figure 7C**). The increased glucose uptake could be due to changes in the expression or subcellular localization of glucose transporters. Western blot analysis showed that GLUT4 expression was similar in Arid5b MKO and Arid5b WT GC (**Figure 7D**). To determine if GLUT4 translocation to the plasma membrane was increased in Arid5b MKO skeletal muscles, we performed coimmunofluorescence staining for GLUT4 and dystrophin, a marker of the plasma membrane, followed by confocal microscopy. This coimmunofluorescence analysis showed increased GLUT4 membrane localization in Arid5b MKO GC compared to Arid5b WT GC (**Figure 8**) and in Arid5b MKO Sol relative to Arid5b WT Sol (**Supplementary Figure 5**). These data suggest that glucose uptake is enhanced due to increased cell surface GLUT4, which is consistent with the increased glucose uptake during the GTT.

**Figure 9:**
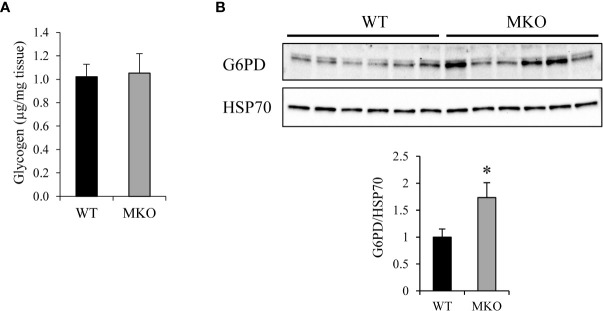
Glucose 6-phosphate dehydrogenase (G6PD) expression was upregulated in Arid5b MKO GC. **(A)** Glycogen content was measured in Arid5b WT and MKO GC. (n=5). Data are presented as the means ± SE. **(B)** G6PD expression was analyzed by Western blot in Arid5b WT and MKO GC and normalized to HSP70 expression. Quantitation was performed, and results are expressed as the means ± SE. *, p < 0.05.

Once taken up into the skeletal muscle, glucose can be stored as glycogen or diverted to the pentose phosphate pathway for generation of 5-carbon sugars for ribonucleotide synthesis and NADPH for lipid synthesis and to reduce levels of reactive oxygen species (ROS). We found that glycogen levels were similar in Arid5b WT and MKO GC (**Figure 9A**). To determine if the pentose phosphate pathway was upregulated in Arid5b MKO muscle, we performed western blot analysis for glucose 6-phosphate dehydrogenase (G6PD), the rate-limiting enzyme in the pathway. We found that G6PD expression was increased in Arid5b MKO GC relative to WT (**Figure 9B**), indicating that glucose flux into the pentose phosphate pathway is increased.

**Figure 10:**
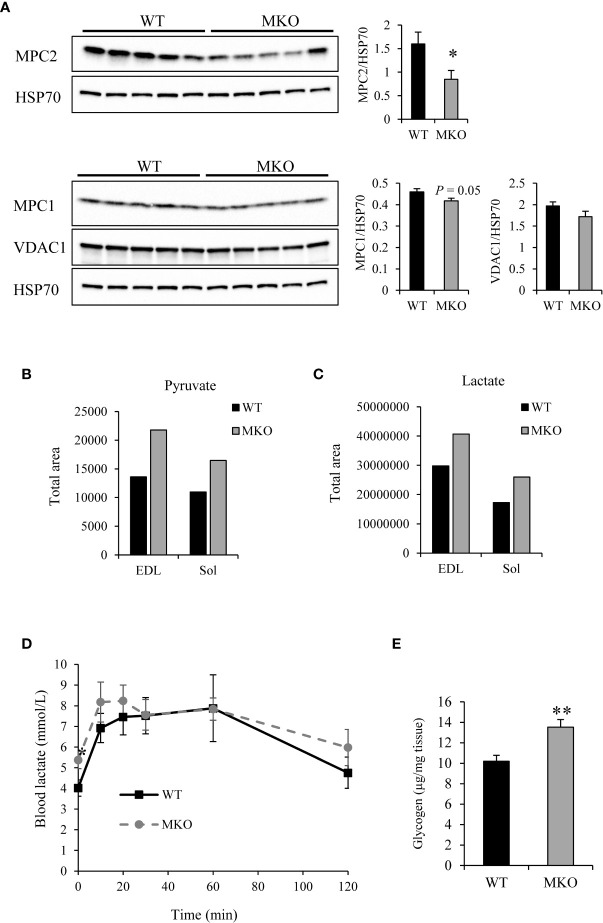
Cori cycling was increased in Arid5b MKO mice. **(A)** Expression of MPC2, MPC1 and VDAC1 were analyzed by Western blot in Arid5b WT and MKO Sol and normalized to HSP70 expression. Quantitation was performed, and results are expressed as the means ± SE. Quantitative mass spectrometry was performed for **(B)** pyruvate and **(C)** lactate in Arid5b WT and MKO muscles. **(D)** Blood lactate was measured during glucose tolerance tests (n=5). *, p < 0.05. **(E)** Glycogen levels were measured in the liver (n=5). Data are presented as the means ± SE. **, p < 0.01.

Recent publications have shown that knockout of the mitochondrial pyruvate carrier (MPC) in skeletal muscle leads to increased glucose uptake and conversion of pyruvate to lactate for export to the circulation, thereby increasing Cori cycling and fatty acid oxidation (30). We therefore evaluated MPC expression levels in Arid5b MKO skeletal muscle. Western blot analysis showed a significant reduction in MPC2 expression levels and a small but significant decrease in MPC1 expression levels in Arid5b MKO Sol relative to WT Sol (**Figure 10A**). Expression of VDAC1, which imports pyruvate from the cytosol into the mitochondrial intermembrane space, was trending lower in Arid5b MKO Sol but did not reach significance. Collectively, our data suggest that in Arid5b MKO skeletal muscle the downregulation of MPC prevents the entry of pyruvate into the mitochondria. Interestingly, targeted mass spectrometry analysis revealed that pyruvate and lactate levels in the Arid5b MKO skeletal muscle were increased (**Figures 10B, C**). Expression of lactate dehydrogenase was similar in Arid5b WT and MKO GC by Western blot analysis (**Supplementary Figure 6**). In addition, lactate levels in blood samples collected for GTT analysis were increased significantly at time 0 and were trending higher during the first 30 min and from 60 to 120 min in Arid5b MKO mice relative to Arid5b WT mice (**Figure 10D**), indicating that the increased lactate is being exported to the bloodstream. Excess lactate from the circulation can be taken up by the liver for gluconeogenesis. While liver weights were similar between Arid5b WT and MKO mice (**Supplementary Figure 7**), glycogen levels in the liver were significantly higher in Arid5b MKO mice relative to Arid5b WT mice (**Figure 10E**), suggesting that the lactate taken up by the Arid5b MKO liver is converted to glycogen. Interestingly, these results indicate that Cori cycling is increased in Arid5b MKO mice.

## Discussion

In this report, we characterized the changes in metabolism in the skeletal muscle of Arid5b MKO mice. We found that Arid5b MKO skeletal muscle preferentially utilizes fatty acids for energy generation. Increased expression of phospho-HSL and HSL in adipose tissue suggest that there may be increased lipolysis to fuel the elevated fatty acid metabolism in skeletal muscle. The reduction in free fatty acids in the plasma along with increased muscle CD36 and FABP4 expression suggests that Arid5b MKO skeletal muscle takes up more fatty acids from the bloodstream. Glucose oxidation in Arid5b MKO skeletal muscle was reduced and was associated with a decrease in MPC expression. Interestingly, glucose uptake was enhanced in Arid5b MKO skeletal muscle with a corresponding increase in GLUT4 translocation to the plasma membrane. Imported glucose was channeled to the pentose phosphate pathway as well as converted to lactate for export to the liver. Taken together, our data suggest that Arid5b MKO skeletal muscle favors fatty acids as the fuel source, influencing metabolism in adipose tissue and liver.

Our results are consistent with the phenotype reported for skeletal muscle-specific deletion of *MPC1* (MPC SkmKO) (30). MPC1 forms a complex with MPC2 on the inner mitochondrial membrane, allowing import of pyruvate into the mitochondrial matrix for oxidation. Disruption of the MPC complex in MPC SkmKO muscle prevented mitochondrial pyruvate uptake, and therefore pyruvate oxidation was decreased. MPC SkmKO mice showed increased skeletal muscle FAO and reduced fat mass relative to WT mice with no change in lean mass. In addition, muscle glucose uptake increased along with lactate excretion, thereby fueling the Cori cycle. In Arid5b MKO mice, MPC2 expression was significantly downregulated and the reduction in MPC1 expression was smaller but reached significance. Accordingly, we observed a similar pattern of metabolic changes as in the MPC1 SkmKO mice, indicating that MPC contributes mechanistically to the metabolic changes observed in Arid5b MKO skeletal muscle. Future studies will investigate the detailed mechanism of perturbation of MPC expression in Arid5b MKO skeletal muscle.

Deletion of Arid5b in the skeletal muscle increased the production of lactate and its export to the circulation. Exported lactate is taken up by the liver and converted to glucose, which can be released back into the bloodstream for uptake by muscle or stored as glycogen in the liver. This Cori cycling is energetically futile since 2 molecules of ATP are produced by glycolysis in the muscle, but 6 ATP are consumed by gluconeogenesis in the liver. Therefore, to support ATP demand, fatty acid oxidation is increased in the skeletal muscle, and previous studies have shown that Cori cycling increases fatty acid oxidation in the liver (31, 32). Since we did not observe a change in food consumption between Arid5b WT and MKO mice, the downregulation of MPC gene expression may require the skeletal muscle to utilize and oxidize fatty acids as the source of energy. Additionally, the increased requirement for fatty acids could potentially lead to the decrease in adipose tissue weight, contributing to the reduction in body weight in the Arid5b MKO mice.

In agreement with the increased fatty acid oxidation in Arid5b MKO skeletal muscle, we observed increases in the expression of the fatty acid transporters CD36 and FABP4. CD36 is one of the principal transporters of fatty acids into the cell. Increased expression of the CD36 transporter could potentially be the cause of the reduced concentration of free fatty acids in the plasma along with the increased fatty acid oxidation rates in the skeletal muscle. FABP4 is expressed inside skeletal muscle fibers (33) and has been shown to traffic lipids between intracellular organelles, including mitochondria, the nucleus and the endoplasmic reticulum (34). As a lipid chaperone, FABP4 has a role in processes including oxidation, lipid storage, membrane synthesis, and lipid-mediated transcriptional regulation (24). Interestingly, FABP4 is also expressed in capillary endothelial cells in skeletal muscle, allowing for fatty acid uptake from the circulation into the muscle (35). In Arid5b MKO muscle, the upregulation of FABP4 may be needed to import fatty acids into the muscle and deliver them to the mitochondria for oxidation to support the increased FAO rates. The reduction of free fatty acids in the plasma indicates that uptake of fatty acids from the bloodstream is increased in Arid5b MKO skeletal muscle. FABP4 in capillary endothelial cells and in the muscle may both play a role to enhance fatty acid uptake and metabolism in Arid5b MKO muscle.

Once taken up into the skeletal muscle, glucose has several different fates: storage as glycogen, oxidation in the mitochondria, or conversion to lactate for export into the circulation. Glucose can also enter the pentose phosphate pathway to generate ribose 5-phosphate for nucleotide synthesis and NAPDH for fatty acid synthesis and for protection against damage from reactive oxygen species (ROS) (36). Fatty acid oxidation is known to increase the level of ROS (37). Since fatty acid oxidation was increased in the Arid5b MKO skeletal muscle, there may be increased production of reactive oxygen species. The Arid5b MKO muscle may be diverting glucose into the pentose phosphate pathway to generate more NADPH to combat ROS damage, which is an area that requires further study.

Interestingly, the increase in glucose uptake in Arid5b MKO muscle was independent of changes to Akt phosphorylation, as was observed in MPC SkmKO mice. These results indicate that glucose uptake is increased in an insulin-independent manner. Several alternative mechanisms of GLUT4 translocation have been reported, including G_q_-coupled GPCR signaling (38), β-adrenergic receptor activation (39), nitric oxide synthase (40), and dietary components (41). Future studies will explore the involvement of these pathways in GLUT4 translocation in Arid5b MKO muscles.

Based on the body weight analysis in which we see a consistent weight difference in the MKO mice from week 6 until week 24, we believe the metabolic changes observed in the Arid5b MKO mice may be an adaptive response to the deletion of *Arid5b*. The increased fatty acid oxidation in Arid5b MKO muscles along with the potential increase in lipolysis in adipose tissue can be enhanced through stimuli such as high fat diet (HFD) or exercise. The MKO mice may be protected from weight gain on the HFD through enhancement of adipose tissue lipolysis and release of FFA due to the demand of the skeletal muscle. Because the MKO mice are leaner on the normal chow diet, exercise may increase the skeletal muscle demand for fatty acid as fuel and enhance the leanness of these mice. The increased fatty acid requirements of the MKO skeletal muscle may lead to increased endurance during exercise.

Crosstalk between skeletal muscle, adipose tissue and liver is important for maintaining energy homeostasis and proper organ function. Our data show that knockout of Arid5b MKO in skeletal muscle led to alterations in the utilization of glucose and fatty acids, favoring fatty acids for energy generation. Interestingly, these changes in skeletal muscle fuel consumption influenced metabolism in adipose tissue and liver. Our results suggest that Arid5b may be a potential target for the treatment of metabolic diseases, including diabetes and obesity.

## Limitations

Due to the lack of availability of a mouse specific antibody for detection of Arid5b protein, we were limited to detection of Arid5b at the mRNA level by real-time PCR. Also, although we have ruled out two major mechanisms for increased mitochondrial biogenesis in the Arid5b MKO muscles as the cause of increased fatty acid oxidation, there are other mechanisms that could be influencing mitochondrial biogenesis and capacity. These mechanisms could involve altered expression or function of PGC1β, calcium/calmodulin-dependent protein kinase II (CamKII), PPARδ, sarcolipin, or estrogen-related receptor α (ERRα).

## Data availability statement

The raw data supporting the conclusions of this article will be made available by the authors, without undue reservation.

## Ethics statement

The animal study was reviewed and approved by City of Hope Institutional Animal Care and Use Committee.

## Author contributions

JM designed the study, carried out experiments, analyzed the data, and wrote the manuscript. AE designed the study, carried out experiments, and critically revised the manuscript. LN and GZ carried out experiments. KI interpreted the data and critically reviewed the manuscript. All authors contributed to the article and approved the submitted version.
