# Supplementary material for: Muscle-specific deletion of Arid5b causes metabolic changes in skeletal muscle that affect adipose tissue and liver
Source: Front Endocrinol (Lausanne). 2023 Jan 18;13:1083311. doi: 10.3389/fendo.2022.1083311 (PMC9891308; doi:10.3389/fendo.2022.1083311)
Supplement: Supplementary file 1 [file Presentation_1.pdf]

Supplementary Figure 1

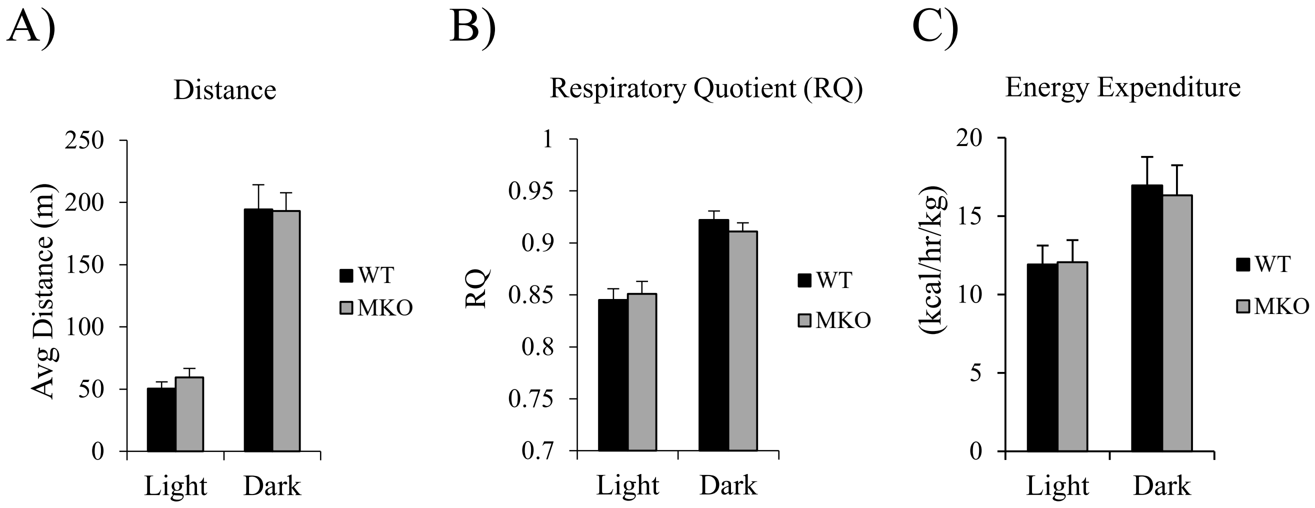

Supplementary Figure 1. The average distance traveled (A), respiratory quotient (RQ) (B), and energy expenditure (C) were analyzed in Arid5b WT and MKO mice (n=8). Data are presented as the means ± SE.

Supplementary Figure 2

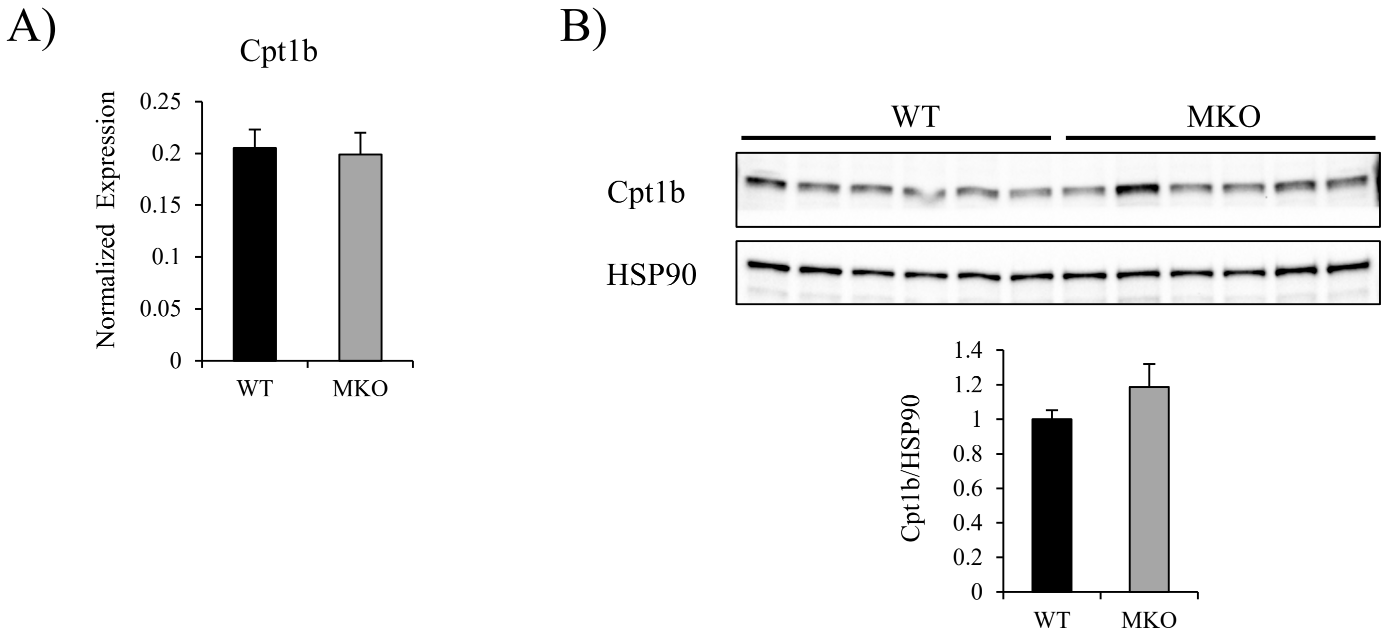

Supplementary Figure 2. Cpt1b expression was similar in Arid5b WT and MKO skeletal muscle. A) Cpt1b expression was analyzed by real-time PCR in GC and normalized to Rpl19 expression. Samples were analyzed in triplicate, and results are expressed as the means ± SE (n=8). B) Western blot analysis was performed for Cpt1b expression in GC muscles and normalized to HSP90 expression. Quantitation of Cpt1b expression was performed, and data are presented as the means ± SE.

Supplementary Figure 3

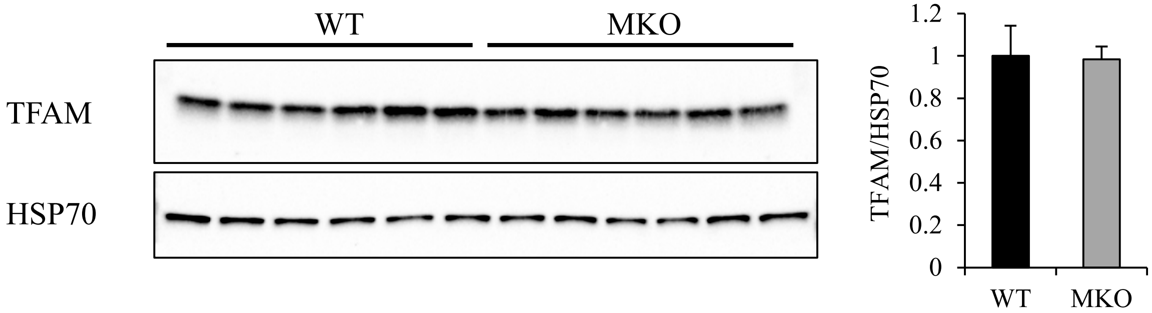

Supplementary Figure 3. Expression of TFAM was similar in Arid5b WT and MKO skeletal muscle. Western blot analysis was performed for TFAM expression in GC muscles and normalized to HSP70 expression. Quantitation of TFAM expression was performed, and data are presented as the means ± SE.

Supplementary Figure 4

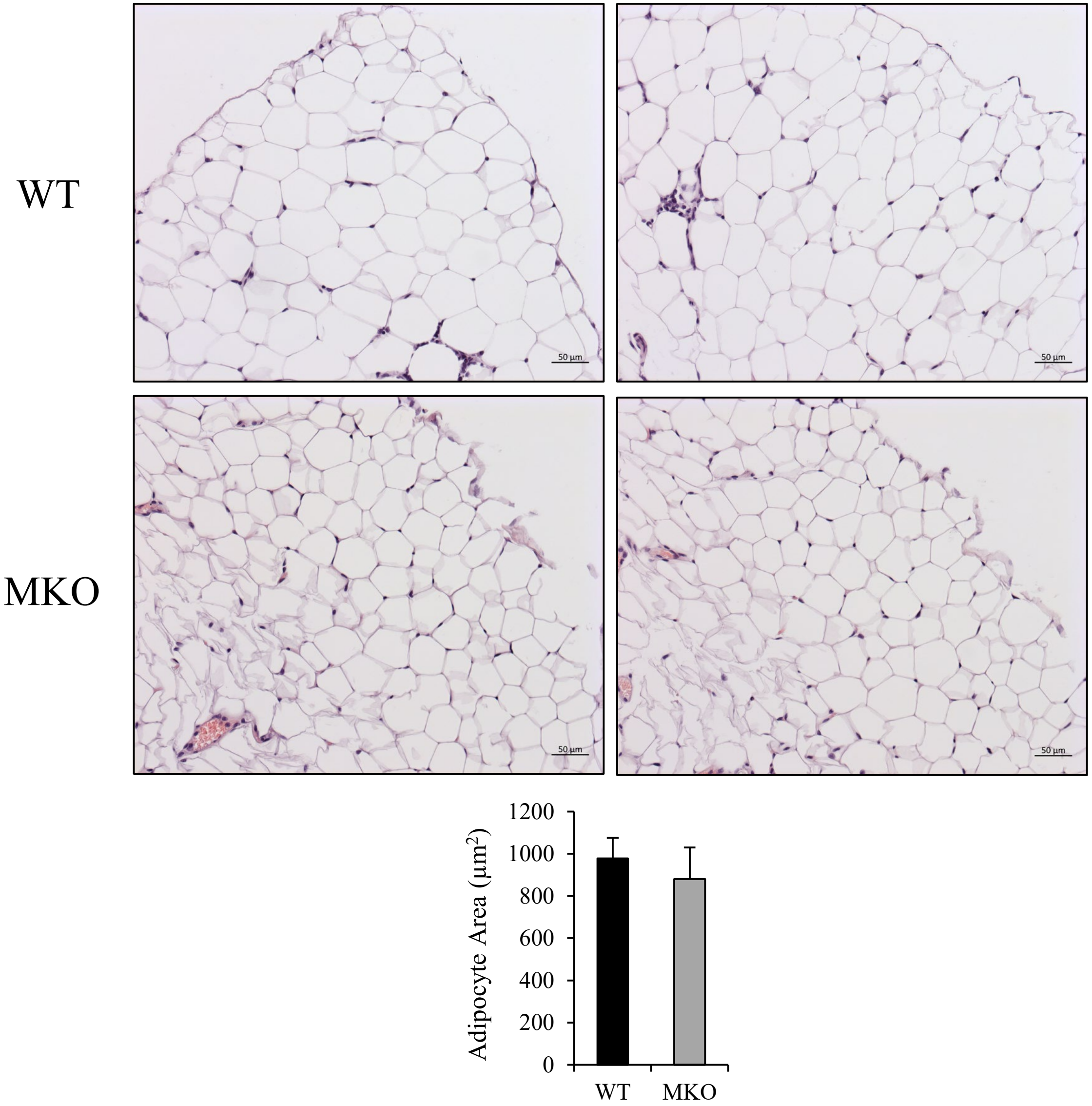

Supplementary Figure 4. H&E staining of GWAT tissue sections from Arid5b WT and MKO at 20x magnification. Scale bar = 50µm. Representative images are shown (n=4). Quantitation of adipocyte size was performed, and results are presented as the means ± SE.

Supplementary Figure 5

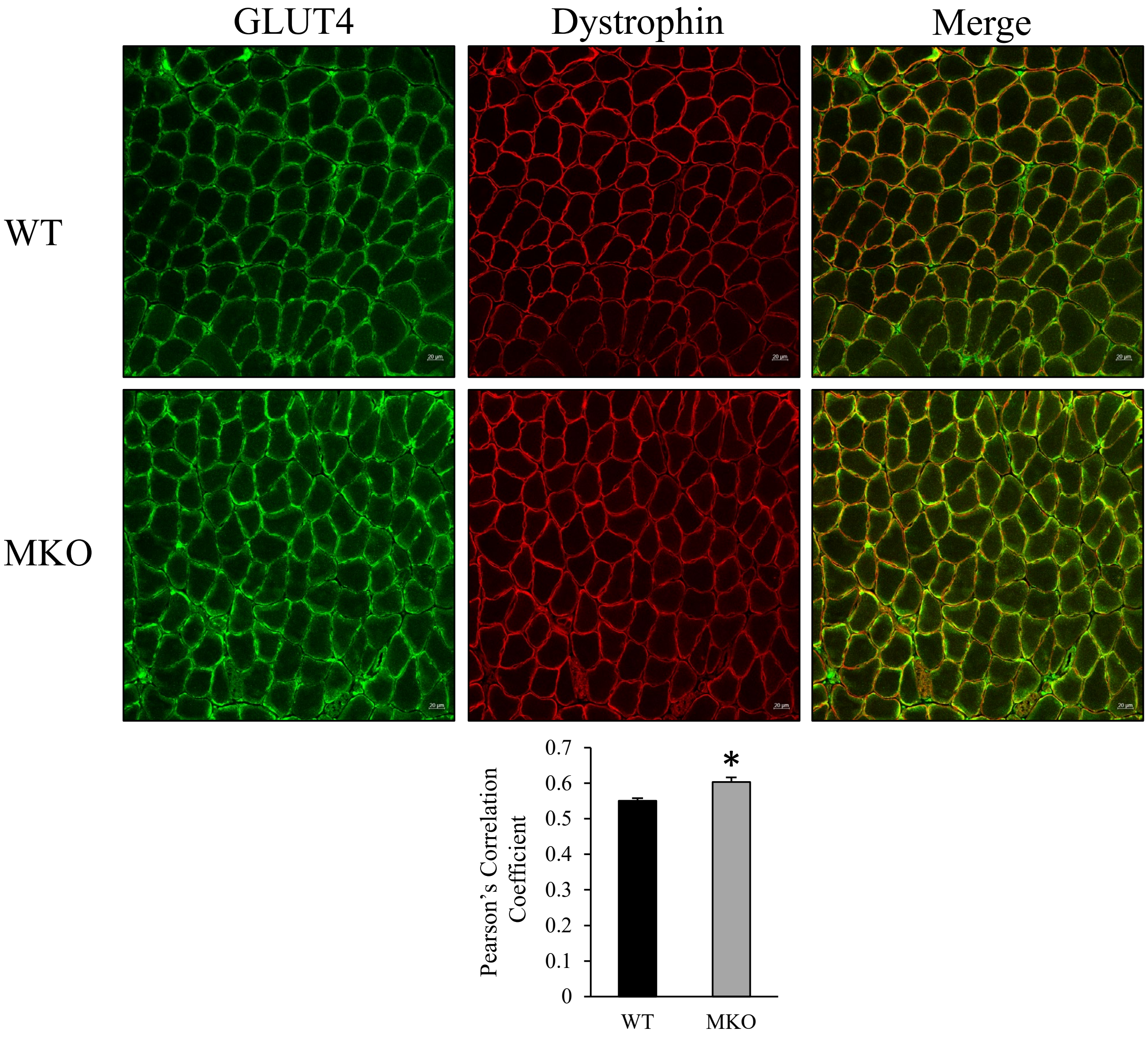

Supplementary Figure 5. GLUT4 localization to the plasma membrane was increased in Arid5b MKO Sol. Coimmunofluorescence analysis for GLUT4 (green) and dystrophin (red) was carried out in Sol tissue sections. Merged images show increased colocalization of GLUT4 and dystrophin at the plasma membrane in Arid5b MKO Sol relative to WT Sol. Representative confocal images are shown. Scale bars = 20µm. Pearson's correlation coefficient was calculated, and data are presented as the means ± SE. *, $p < 0.05$. (n=4).

Supplementary Figure 6

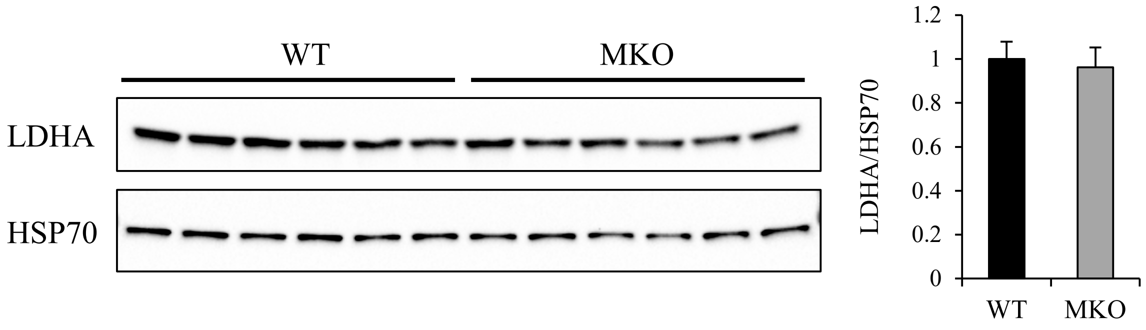

Supplementary Figure 6. Expression of lactate dehydrogenase A (LDHA) was analyzed by Western blot in Arid5b WT and MKO GC. HSP70 was included as a loading control. Quantitation was performed, and results are expressed as the means ± SE.

Supplementary Figure 7

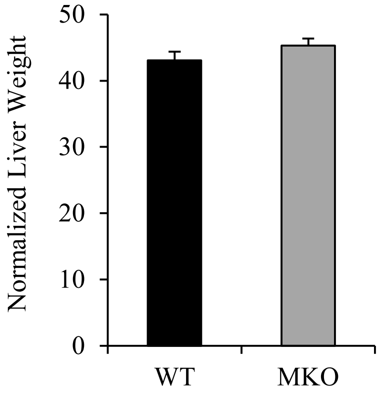

Supplementary Figure 7. Liver weights were normalized to body weights (n=14). Data are presented as the means ± SE.
